# Ubiquilin 2 Is Not Associated with Tau Pathology

**DOI:** 10.1371/journal.pone.0076598

**Published:** 2013-09-26

**Authors:** Anna Nölle, Elise S. van Haastert, Rob Zwart, Jeroen J. M. Hoozemans, Wiep Scheper

**Affiliations:** 1 Department of Genome Analysis, Academic Medical Center/University of Amsterdam, Amsterdam, The Netherlands; 2 Department of Pathology, VU University Medical Center, Amsterdam, The Netherlands; 3 Department of Neurology, Academic Medical Center/University of Amsterdam, Amsterdam, The Netherlands; Hertie Institute for Clinical Brain Research and German Center for Neurodegenerative Diseases, Germany

## Abstract

Accumulation of aberrant proteins in inclusion bodies is a hallmark of many neurodegenerative diseases. Impairment of proteolytic systems is a common event in these protein misfolding diseases. Recently, mutations in the *UBQLN 2* gene encoding ubiquilin 2 have been identified in X-linked amyotrophic lateral sclerosis (ALS). Furthermore, ubiquilin 2 is associated with inclusions in familial and sporadic ALS/dementia, synucleinopathies and polyglutamine diseases. Ubiquilin 2 exerts a regulatory role in proteostasis and thus it has been suggested that ubiquilin 2 pathology may be a common event in neurodegenerative diseases. Tauopathies, a heterogenous group of neurodegenerative diseases accompanied with dementia, are characterized by inclusions of the microtubule-binding protein tau. In the present study, we investigate whether ubiquilin 2 is connected with tau pathology in Alzheimer’s disease (AD), supranuclear palsy (PSP) and Pick’s disease (PiD) and familial cases with frontotemporal dementia and parkinsonism linked to chromosome 17 (FTDP-17). We show that ubiquilin 2 positive inclusions are absent in these tauopathies. Furthermore, we find decreased ubiquilin 2 protein levels in AD patients, but our results do not indicate a correlation with tau pathology. Our data show no evidence for involvement of ubiquilin 2 and indicate that other mechanisms underly the proteostatic disturbances in tauopathies.

## Introduction

Aggregation and accumulation of aberrant proteins is a common event in neurodegenerative diseases. Neurons are post-mitotic cells that are highly dependent on efficient protein quality control systems to ensure protein homeostasis. This includes protein folding, recognition and correction of misfolded proteins as well as degradation of terminally misfolded proteins by the proteolytic machinery consisting of the ubiquitin-proteasome system and autophagy/lysosomal pathway [1]. Failure in proteolysis may lead to protein accumulation, build-up of protein intermediates, and ultimately formation of inclusion bodies. Impairment of both degradational pathways is implicated in neurodegeneration and thus provides a potential target for therapeutic intervention. Recently, the ubiquitin-binding protein ubiquilin 2 has been linked to amyotrophic lateral sclerosis (ALS), a rapidly progressive motoneuron disease, which is in about 20% of patients accompanied by frontotemporal dementia [2]. Deng et al. identified missense mutations in *UBQLN 2* gene in familial cases of dominant X-linked ALS and ALS/dementia. Furthermore, using immunohistochemistry the group reported that mutant ubiquilin 2 accumulates in neuronal inclusions in the spinal cord and brain. Ubiquilin 2 positive inclusions were also found in sporadic cases of ALS and ALS/dementia, which together present approximately 90% of the patients indicating a causative role for ubiquilin 2 in a broad spectrum of ALS subtypes. Strikingly, ubiquilin 2 pathology in the hippocampus of patients with familial and sporadic ALS was correlated with dementia [2]. In addition, ubiquilin 2 pathology was found in association with inclusion bodies in synucleinopathies and polyglutamine diseases as well [3,4]. It was therefore suggested that ubiquilin 2 pathology may be a general pathomechanism in neurodegenerative diseases.

Ubiquilins are ubiquitin-binding proteins that play a regulatory role in proteolysis. The human genome encodes four ubiquilins, which share the N-terminal ubiquitin like domain (UBL) and C-terminal ubiquitin-associated domain (UBA). This specific structure facilitates delivery of poly-ubiquitinated proteins to the proteasome [5]. The conserved UBL and UBA domains are flanking a more variable central region containing several Sti1 repeats, which confer chaperone-like activity [6]. Various interaction partners are known for ubiquilin 1, the most studied protein of the ubiquilin family, which was originally identified in a yeast two-hybrid screen as an interactor with the AD associated protein presenilin 1 and 2 [7]. Ubiquilin 2 shows 79% homology with ubiquilin 1, and although *UBQLN 2* is less ubiquitously expressed than *UBQLN 1*, it can be assumed that various functions discovered for the ubiquilin 1 also apply to ubiquilin 2 [7,8].

Ubiquilin 1 is reported to be involved in endoplasmic reticulum (ER)-associated degradation (ERAD), a mechanism by which aberrant proteins are translocated from the ER into the cytosol for degradation by the proteasome [9]. Ubiquilin 1 levels are increased during ER stress and overexpression enhances viability, which may be mediated by interaction with the ERAD complex. Another line of study shows that ubiquilin 1 protects from starvation-induced cell death via (macro) autophagy [10,11]. Autophagy involves formation of double membrane vesicles known as autophagosomes, which subsequentially fuse with the lysosome for protein degradation. Ubiquilin is associated with the autophagy marker LC3, which is a constituent of the autophagosome [10]. Furthermore, ubiquilin 1 interferes with mTOR, the inhibition of which induces autophagy [12].

Several single nucleotide polymorphisms of the *UBQLN 1* gene have been linked to increased risk to develop Alzheimer’s disease (AD) pathology [13,14]. In addition, a decrease of ubiquilin 1 protein levels in the brain of late onset AD patients with Braak stage 3-6 has been observed [15]. AD is characterized by intracellular inclusions of the microtubule binding protein tau which are also present in various other neurodegenerative diseases associated with dementia, referred to as tauopathies. AD, the most prevalent tauopathy, is characterized by neurofibrillary tangles (NFTs) comprised of hyperphosphorylated tau and extracellular deposits of amyloid-β (Aβ). Loss of ubiquilin 1 function may contribute to Aβ pathology in AD [15-17].

In tauopathies such as Pick’s disease (PiD), progressive supranuclear palsy (PSP) and frontotemporal dementia and parkinsonism linked to chromosome 17 (FTDP-17) the presence of tau inclusions is the sole pathological hallmark. Tau inclusion bodies vary in shape, affected cell type and brain regions leading to specific clinical symptoms in these diseases [18].

The role of ubiquilin 2 in proteolysis suggests that ubiquilin 2 pathology may be a general event in protein misfolding diseases. Here, we studied whether ubiquilin 2 is associated with tau pathology using post-mortem material from tauopathy patients.

**Table 1:** Cases used in this study.

**Case**	**n**	**Age**	**Braak stage**
**Single staining**			
con	4	57-74	0-3
AD	6	54-87	4-6
PiD	3	41-70	-
MAPT mutation carrier	5	49-68	-
PSP	2	67,72	-
**Double staining**			
con	1	66	-
AD	8	64-87	5-6
PiD	5	54-82	-
MAPT mutation carrier	1	51	-
**WB**			
con	7	41-84	1
AD	7	72-85	4-6
**qPCR**			
con	11	80-93	1-3
AD	11	75-94	3-6

con, control; AD, Alzheimer’s disease; MAPT, microtubule associated protein tau; PiD, Pick’s disease; PSP, progressive supranuclear palsy; WB, Western blot; qPCR, quantitative PCR.

## Materials and Methods

### Cell culture

Human SK-N-SH neuroblastoma cells (European Collection of Cell Cultures #86012802, Salisbury, UK) were cultured in Dulbecco’s modified Eagle’s medium with GlutaMax (Gibco BRL, Carlsbad, CA) supplemented with 10% fetal calf serum (Lonza, Basel, Switzerland) and 100 U/ml penicillin (Yamanouchi Pharma BV, Leiderdorp, the Netherlands). Cells were differentiated in culture medium supplemented with all trans-retinoic acid (Sigma, St Louis, MO, USA) in a final concentration of 10 µM for 5 days. Differentiated cells were subsequently treated with tunicamycin(TM) or 2-deoxyglucose (2-DG) at indicated concentrations for 16 h.

**Figure 1:**
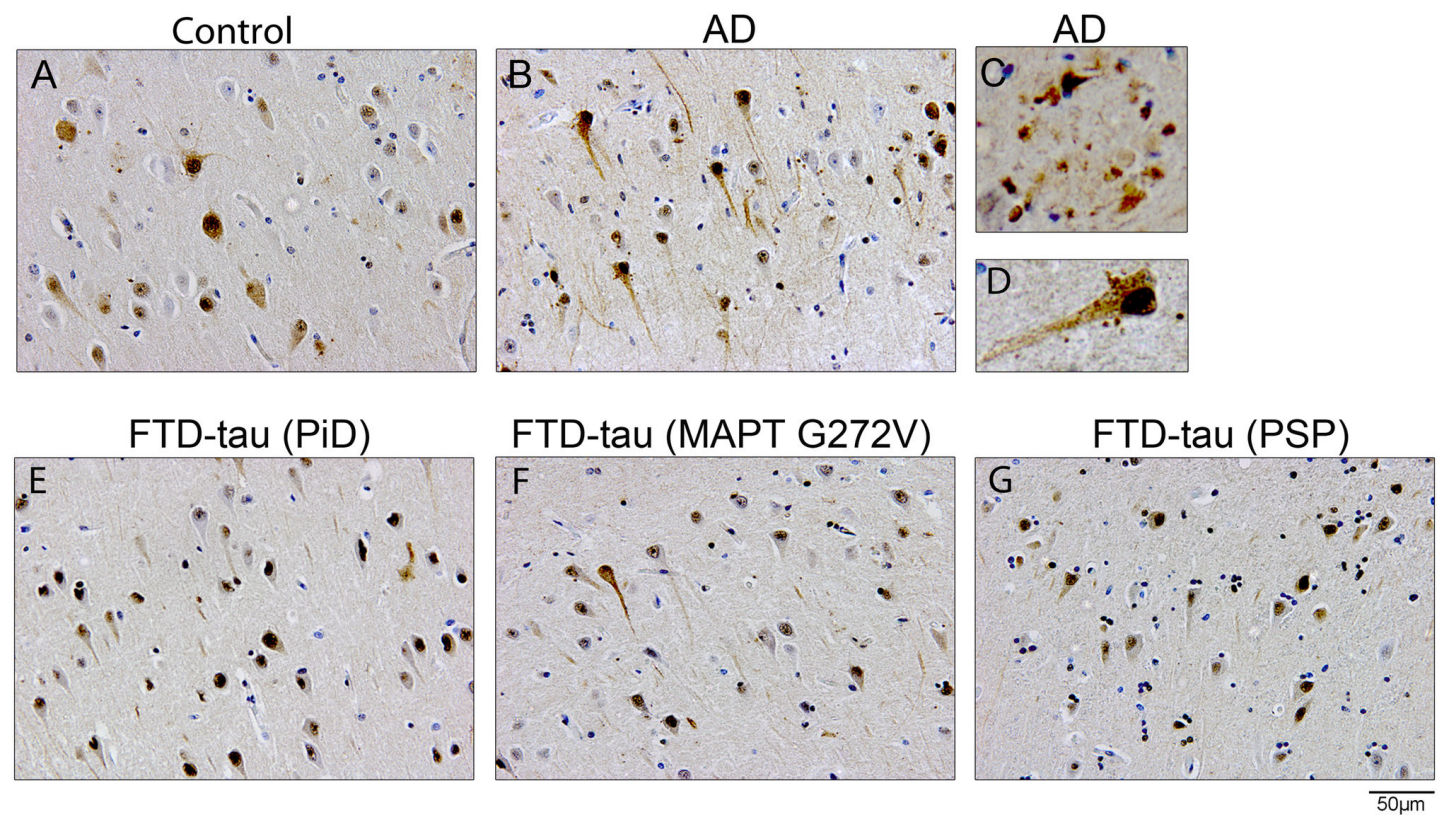
Ubiquilin 2 immunoreactivity in tauopathies. Representative immunohistochemical staining of ubiquilin 2 in the CA1 region of the hippocampus in a control (A) and an AD case (B). Detailed images showing plaque-like structures (C) and perisomatic granules (D) in an AD case. Ubiquilin 2 immunoreactivity in the subiculum of a sporadic PiD case (E), a G272V MAPT mutation carrier (F), and a PSP case (G). AD, Alzheimer’s disease; CA, cornu ammonis, FTD, frontotemporal dementia; MAPT, microtubule associated protein tau.

### Postmortem material

Post-mortem brain material was obtained from the Netherlands Brain Bank (Amsterdam, The Netherlands). Patients or their next of kin gave informed consent for brain autopsy and use of tissue and medical records for research purposes. Staging of AD pathology was evaluated according to Braak and Braak [19]. Age, gender, clinical diagnosis, Braak score for NFTs and post-mortem interval of the cases used in this study are listed in Tables S1, S2, S3 and S4.

### Immunohistochemistry

Formalin-fixed, paraffin-embedded tissue was selected from age-matched non-neurological controls, AD and tau-FTD cases (Table 1). Sections (5 µm thick) were mounted on Superfrost plus tissue slides (Menzel-Glaser, Germany) and dried overnight at 37°C. After deparaffinizing, endogenous peroxidase activity was quenched using 0.3% H_2_O_2_ in Phosphate-buffered saline (PBS) for 30 min. Sections were treated in 10 mM pH 6.0 sodium citrate buffer heated by autoclave during 10 min for antigen retrieval. Sections were washed with PBS and incubated with ubiquilin 2 antibody (Novus Biologicals, Littleton, CO, USA) diluted in Dako antibody diluent (1:10 000, Dako, Hamburg, Germany) for 1 h at room temperature. Negative controls were generated by omission of primary antibodies. After washing with PBS sections were incubated with horseradish peroxidase-labelled mouse/rabbit secondary antibody (REAL EnVision/HRP Rabbit/Mouse, Dako) for 30 min at room temperature. After another washing step color was developed using 3,3′-diaminobenzidine (DAB, Sigma) as chromogen for 10 min. Sections were counterstained with hematoxylin and mounted using Depex (BDH Laboratories Supplies, East Grinstead, UK). For double staining with AT8 (tau pSer202/Thr205, Thermo, Fisher Scientific, Rockford, IL) sections were treated as described above for ubiquilin 2 staining. After developing with 3,3′-diaminobenzidine sections were washed and subsequently treated with 10 mmol/l pH 6.0 sodium citrate buffer heated by autoclave for 10min to denature bound antibodies. After short washing in PBS, sections were blocked with 10% normal goat serum (Dako) for 10 min at room temperature. Incubation with AT8 antibody (1:100 in antibody diluent) was performed overnight at 4 °C. After washing sections were incubated with HRP-conjugated goat anti-mouse IgG1 (1:100 in antibody diluent) for 1 h. Color was developed using Liquid Permanent Red (LPR; DAKO). Sections were counterstained with hematoxylin and mounted using aquamount (BDH Laboratories Supplies). Images were taken with the same microscope settings using a 20 x objective. At least two different fields of view were analyzed per patient. Ubiquilin 2 and AT8 signals were unmixed using Nuance spectral imaging system (CRi, Woburn, MA, USA) as described previously [20]. AT8 and ubiquilin 2 double stained sections of 7 AD cases (Table 1) were used to compare ubiquilin 2 signals between AT8 positive and AT8 negative cells. Numbers of AT8 positive and AT8 negative cells, respectively, were determined and assigned to the respective group. To determine neurons displaying a high ubiquilin 2 signal in these groups, a threshold for ubiquilin 2 intensity was set using ImageJ software. The threshold was changed for two sections (case 4, 7) because of a variation in staining intensity. Neurons, under and above the threshold, respectively, were counted and assigned to ubiquilin 2 low or ubiquilin 2 high group. Subsequently, frequency distributions were compared between AT8 positive and AT8 negative groups using the chi-square test. A p value of <0.05 was taken as significant.

**Figure 2:**
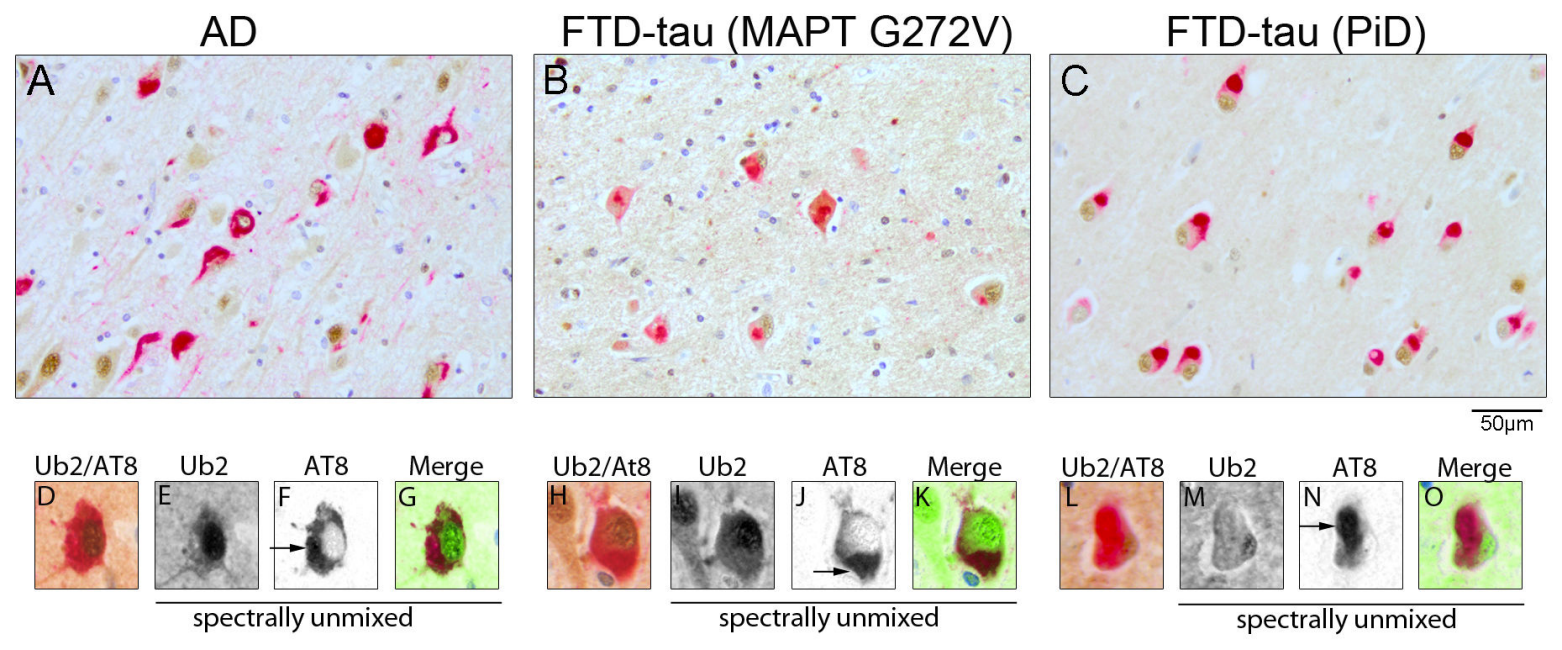
Tau inclusions are not immunoreactive for ubiquilin 2. Double immunostaining for ubiquilin 2 and AT8 in an AD case (A), a G272V MAPT mutation carrier (B) and a sporadic PiD case (C). Detailed images of double labeling show neurons with AT8-positive tau inclusions (D-O). Images were spectrally unmixed, and shown separately and merged in artificial colors (ubiquilin 2: green; AT8: red). AD, Alzheimer’s disease; CA, cornu ammonis FTD, frontotemporal dementia; MAPT, microtubule-associated protein tau, Ub2, ubiquilin 2.

### Western Blot

Brain material (Table 1) was homogenized in lysis buffer (150 mM NaCl, 50 mM Tris HCl pH 7.6, 1% NP-40, 0.5% Sodium Deoxycholate, 0.1% SDS, 2 mM EDTA) supplemented with protease inhibitors (Complete protease inhibitor cocktail from Roche, Penzberg, Germany) and phosphatase inhibitors (PhosSTOP, Roche). Lysates were incubated for 30 min at 4°C and centrifuged for 20 min at 20.000 × g. For analysis of insoluble proteins, pellets were resuspended in 70% formic acid, incubated for 30 min at room temperature and lyophilized using a SpeedVac concentrator. Proteins were resolved in PBS and loaded in a 10% SDS gel. Protein concentrations of the supernatants were determined using Bicinchoninic acid (BCA) assay reagent (Thermo, Fisher Scientific, Rockford, IL, USA). Equal amounts of protein were loaded on a 10% SDS gel and analysis was performed as described previously [20]. Ubiquilin 2 antibody (Novus Biologicals) and GAPDH (Millipore Corporation, Billerica, MA, USA) antibody, respectively, were employed in a 1:1000 dilution. GraphPad Prism Sotfware was used for statistical analysis of the data. Mann-Whitney test was used to test for differences between groups. A p value of <0.05 was taken as significant.

**Figure 3:**
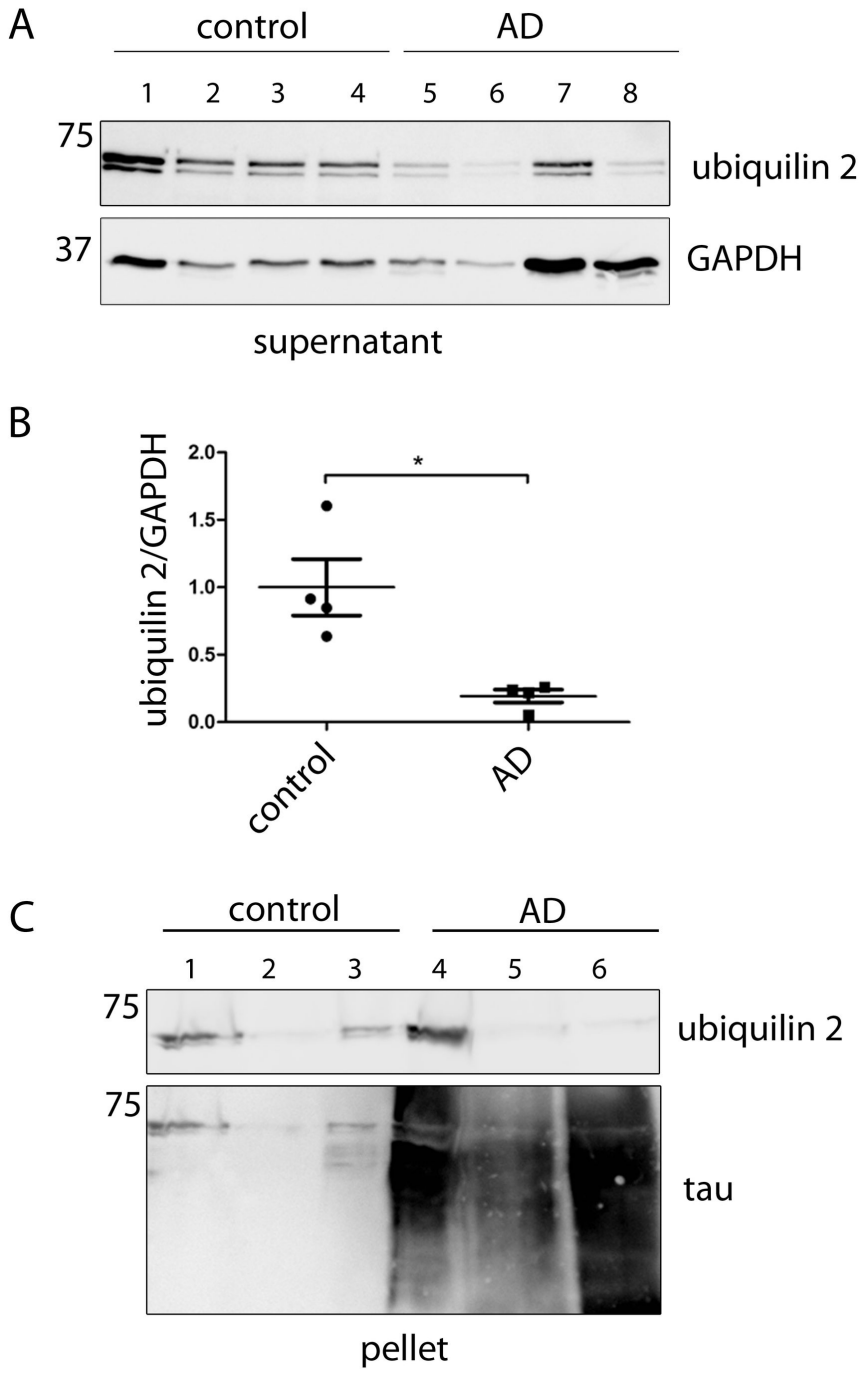
Ubiquilin 2 levels are decreased in AD patients. (A) Western blot analysis of brain lysates from AD (Braak stage 5-6) and control (Braak stage 1) brain material using an ubiquilin 2 antibody and a GAPDH antibody as loading control. (B) Quantification of Western blot signals in (A). Ubiquilin 2 signals were normalized to GAPDH and statistically analysed (Mann–Whitney test, * p <0.05). (C) Supernatant and pellet lysate fractions of brain material from 3 AD patients (Braak stage 6) and 3 controls (Braak stage 1) were analysed by Western blotting. Pellets were treated with formic acid to dissolve aggregates prior to Western blotting. GAPDH was used as loading control (supernatant); tau was used as a control for successful extraction of the aggregates in the AD samples (pellet). AD, Alzheimer’s disease.

**Table 2:** Primers and probes used for qPCR.

Gene (human)	Primers (5'–3')	Probe
UBQLN2	FW: gcagcctgaaggatcagtgta	72
	RV: tgggagaagctgagaaggaa	
GAPDH	FW: gctgagtccgcagcagg	45
	RV: tgccaacagggagagcaga	
BiP	FW: catcaagttcttgccgttca	10
	RV: tcttcaggagcaaatgtctttgt	

Probe numbers refer to numbers in the Roche universal probe library.

### RNA isolation and cDNA synthesis

For RNA isolation, brain material (Table 1) or cells were homogenized in TRIzol reagent (Invitrogen, Carlsbad, CA, USA) and total RNA was isolated using the QIAcube (QIAGEN, Venlo, The Netherlands). The protocol was used according to the manufacturer’s specifications. RNA concentrations and purity were assessed by OD measurements at 260 and 280nm on a NanoDrop spectrophotometer (Thermo Scientific). For cDNA synthesis, 1μg of RNA and 125pmol OligodT12 primer were dissolved in a total of 10μl H2O and incubated at 72°C for 10min. Reverse transcriptase mix was added, consisting of 5μl 5 × first-strand buffer (Invitrogen), 0.5μl SuperScript II RNA polymerase (Invitrogen), 10mM dNTPs and 25mM MgCl2 in a total of 15μl H20. The mixture was incubated at 42°C for 1h, followed by 15min at 70°C. cDNA quality was assessed on 0.8% agarose gel.

### Real-time qPCR

Real-time qPCR was performed using the Light Cycler 480 system (Roche Applied Science, Indianapolis, IN, USA). Oligonucleotide primers (Sigma) used for qPCR are listed in table 2. Reaction volumes of 5μl contained cDNA, 0.1μM Universal Probe Library probe (Roche Applied Science), also listed in table 2, 0.4μM forward primer, 0.4μ reverse primer and 2.5μl 2 × LightCycler 480 Probes Master (Roche Applied Science). After denaturation for 10min at 95°C, amplification was performed using 35 cycles of denaturation (95°C for 10s), followed by annealing (58°C for 15s) and elongation (72°C for 15s). Results were analyzed using the LightCycler 480 software (Roche Applied Science) version 1.5. GraphPad Prism Software was used for statistical analysis of the data. Mann-Whitney test was used to test for differences between groups. A p value of <0.05 was taken as significant.

## Results

### Ubiquilin 2 is not associated with inclusions in tauopathies

Deng et al. showed that wt ubiquilin 2 accumulates in pathological inclusions in the spinal cord and brain of ALS/dementia patients [2]. Because ubiquilin 2 associates with different types of protein inclusions, this could indicate a general role for ubiquilin in protein aggregates associated with neurodegeneration. To investigate whether ubiquilin 2 is involved in tau pathology, immunohistochemical analyses were performed on paraffin-embedded brain tissue from tauopathy patients and non-demented controls (Table 1). Hippocampus and temporal cortex are areas with prominent tau pathology in these diseases and were thus selected for the analysis. In all cases, ubiquilin 2 reactivity was diffusely distributed throughout the cytoplasm of granular and pyramidal cells in the hippocampus (Figure 1). A number of neurons displayed a strong nuclear ubiquilin 2 signal. The intensity of ubiquilin 2 immunoreactivity varied among subfields of the hippocampus and areas that displayed a strong ubiquilin 2 signal differed between cases. However, these differences in ubiquilin 2 staining patterns were also observed in control cases and showed no consistent pattern correlating with disease. Ubiquilin 2 reactivity was also found associated with extracellular plaque-like structures and granules resembling perisomatic granules described by Probst et al. [21] and this was predominantly found in AD cases (Figure 1C, D). No intracellular ubiquilin 2 positive inclusions were observed in hippocampal neurons in any of the tauopathy cases. In line with the findings in the hippocampus, ubiquilin 2 was diffusely distributed in neurons in the temporal cortex (Figure S1).

To further investigate the potential role of ubiquilin 2 in tau pathology, hippocampal tissue of eight AD and six FTD patients (Table 1) was double-stained with the AT8 antibody, which specifically detects hyperphosphorylated tau (Figure 2). Consistent with literature, pronounced AT8 immunoreactivity was detected in Cornu ammonis (CA) region 1, CA region 3 and the subiculum in the cohort [19,22]. In Pick’s disease, tau inclusions known as Pick bodies were also present in the granular cells of the dentate gyrus [22]. Spectral unmixing demonstrated that ubiquilin 2 does not co-localize with tau-positive inclusions (Figure 2, D-O) in any of the cases. These results indicate that direct association of ubiquilin 2 with pathological inclusions as described for ALS/dementia using the same antibody is not detected in tauopathies.

**Table 3:** Semi-quantitative analysis of AD cases double-stained with ubiquilin 2 and AT8.

**Number of cells**	**AT8 positive**	**AT8 negative**	**z**	**p value**	**AT8 positive**
**Sum (case 2-8)**					
**Ubiquilin 2 low**	109	227	12.28	0.00045784	Decrease
**Ubiquilin high**	87	110			
**Case 2**					
**Ubiquilin 2 low**	14	30	34.10	5.2351E-09	Decrease
**Ubiquilin high**	26	10			
**Case 3**					
**Ubiquilin 2 low**	29	22	0.10	0.75363717	ns
**Ubiquilin high**	15	13			
**Case 4**					
**Ubiquilin 2 low**	11	44	0.00	1	ns
**Ubiquilin high**	4	18			
**Case 5**					
**Ubiquilin 2 low**	9	33	3.49	0.06190065	ns
**Ubiquilin high**	11	18			
**Case 6**					
**Ubiquilin 2 low**	36	32	31.50	1.9944E-08	Increase
**Ubiquilin high**	6	43			
**Case 7**					
**Ubiquilin 2 low**	4	40	48.43	3.4171E-12	Decrease
**Ubiquilin high**	14	7			
**Case 8**					
**Ubiquilin 2 low**	6	26	106.25	6.4996E-25	Decrease
**Ubiquilin high**	11	1			

Ubiquilin 2 low indicates number of cells with low or no ubiquilin 2 staining, and ubiquilin 2 high indicates number of cells with high ubiquilin 2 staining. Frequency distributions were compared between cells with AT8 staining (AT8 positive) and without AT8 staining (AT8 negative) using a chi-square test. The right column indicates a significant decrease or increase of the number of ubiquilin 2 low cells in the AT8 positive group. ns, non significant.

### Ubiquilin 2 levels are decreased in AD

Lower levels of ubiquilin 1 were previously reported for AD brain [15]. Therefore, ubiquilin 2 protein levels in cortical tissue of four AD patients and controls were determined by Western blotting. The result showed that ubiquilin 2 levels were significantly decreased in AD (Figure 3) The ubiquilin 2 antibody we used detected a weak band below the ubiquilin 2 band, which suggests minor crossreactivity with the 66 kD ubiquilin 1. Since the soluble protein fraction was used in this analysis, we wanted to exclude the possibility that ubiquilin 2 protein was retained by aggregates present in the insoluble protein fraction. Thus, supernatant and pellet fraction from whole cell lysates were examined from three AD and three control cases. Pellets were treated with formic acid to dissolve aggregated proteins prior to Western blot analysis. Consistent with our first analysis ubiquilin 2 was reduced in supernatant fraction of the AD samples (data not shown). A smear of tau positive material was detected in the pellet fraction of AD samples indicating that extraction of proteins from the pellet was successful (Figure 3 C). The ubiquilin 2 levels in the pellet fraction varied between patients, but showed no consistent difference between AD patients and controls. This indicates that ubiquilin 2 is not sequestered in the insoluble fraction, which is supported by the lack of co-localization of ubiquilin 2 immunoreactivity with tau inclusions (Figure 2, D-O). Therefore, our data indicate that the expression of ubiquilin 2 protein is decreased in the brains of AD patients.

**Figure 4:**
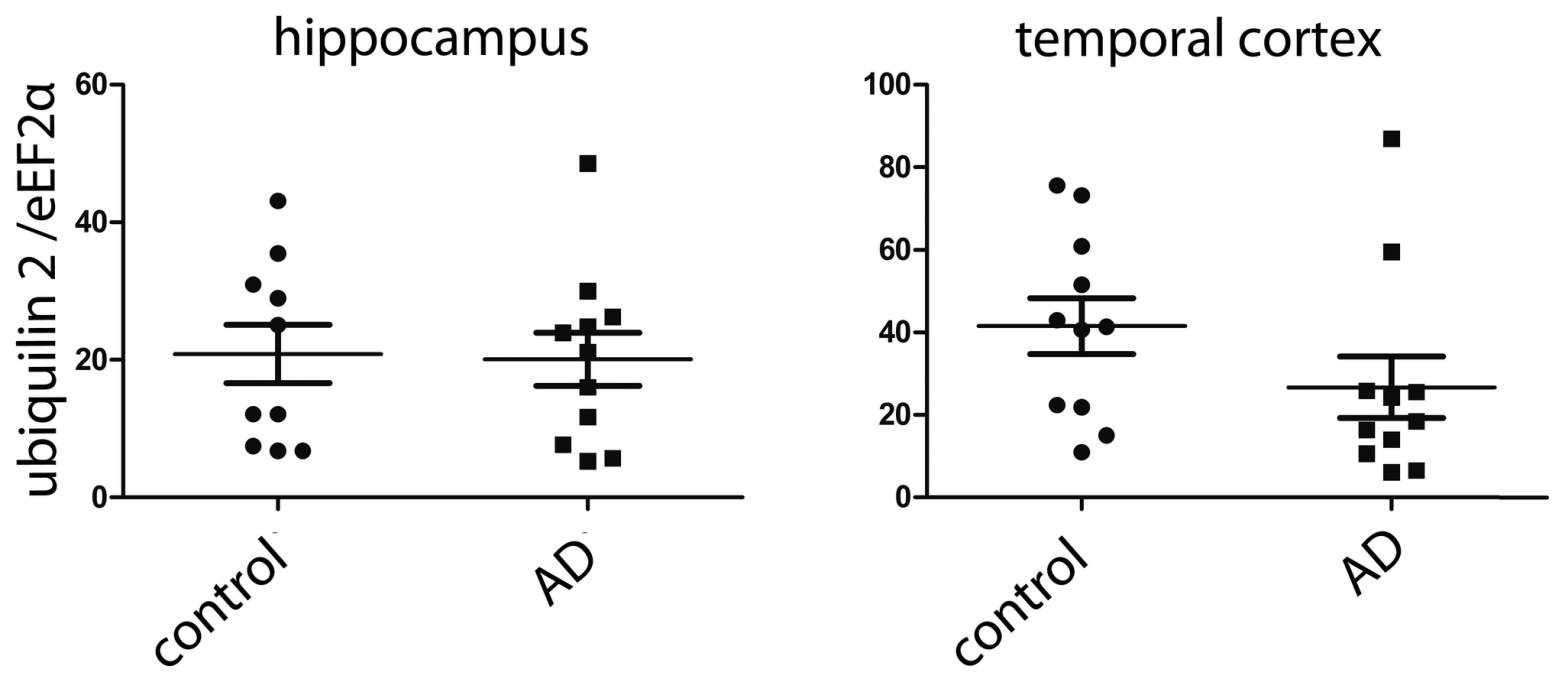
Ubiquilin 2 mRNA levels are not changed in AD. Ubiquilin 2 mRNA levels were assessed by qPCR in hippocampus and temporal cortex from AD and control brain. GAPDH was used as a reference gene (Mann–Whitney test, non-significant).

To investigate the cause of the decrease in protein levels, we analyzed whether the ubiquilin 2 mRNA levels are decreased as well and found no difference between AD and controls, neither in the hippocampus nor in the cortex (Figure 4). This suggests that a post-transcriptional mechanism (decreased translation or increased degradation) is involved in the downregulation of the ubiquilin 2 levels in AD.

### Low ubiquilin 2 levels are not linked to tau pathology

To determine whether ubiquilin 2 levels are selectively lower in cells showing tau pathology, a semi-quantitative analysis of AD cases double-stained with ubiquilin 2 and AT8 was performed (described in detail in material and methods). Briefly, number of neurons with and without AT8 staining, respectively, were determined in the CA1 area of the hippocampus in 7 AD patients and assigned to AT8 positive or AT8 negative group. Subsequently, for each group numbers of cells showing no or low immunoreactivity for ubiquilin 2 (ubiquilin 2 low) and with high immunoreactivity (ubiquilin 2 high), respectively, were counted and frequency distributions were compared between the two groups using a chi-square test (Table 3). Analysis of the total cell numbers from 7 patients showed no association of lower ubiquilin 2 levels with AT8 staining. In contrast, we found that ubiquilin 2 signals are higher in AT8 positive cells. Since the cell numbers counted were different for each patient, we analysed the cases separately and found a high variation in frequency distributions. However, only case 6 showed lower ubiquilin 2 levels in AT8 positive group (Table 3). Clearly, our analysis showed that low ubiquilin 2 levels in AD detected in the Western blot analysis are not associated with the occurrence of tau pathology.

## Discussion

Ubiquilin 2 has recently been linked to intraneuronal inclusions in ALS and ALS/dementia. In the present study, we investigated whether ubiquilin 2 pathology described for ALS/dementia is also present in tau-related dementias (AD and FTD-tau), which are characterized by inclusions comprised of hyperphosphorylated tau. We found no ubiquilin 2 positive inclusions in AD and FTD-tau hippocampus and temporal cortex and conclude that ubiquilin 2 pathology as described for ALS does not occur in tauopathies. Ubiquilin 1 and 2 are highly homologous proteins and there is some crossreaction of the ubiquilin 2 antibody used in this study with ubiquilin 1 (Figure 3). This does not affect the conclusion that ubiquilin 2 does not colocalize with tau inclusions, but suggests that the conclusion may be extended to ubiquilin 1, however this should be confirmed with a ubiquilin 1 antibody. Our findings are supported by the study of Mori et al. reporting ubiquilin 2 pathology in synucleinopathies, polyglutamine diseases and intranuclear inclusions body disease but not in tauopathies [4].

Ubiquilin 2 has been implicated in a variety of proteostatic processes. It is therefore likely to be upregulated in response to proteostatic disturbances. In *C. elegans* ubiquilin 2 is upregulated if the unfolded protein response (UPR) is activated, a stress response aimed to restore disturbed proteostasis in the ER [9]. We have previously shown that the UPR is activated in tauopathies [20,23]. However, in this study we did not detect changes in ubiquilin 2 mRNA levels in AD cortex or hippocampus. Interestingly, we do not observe induction of ubiquilin 2 expression in a human cell model upon UPR activation indicating that ubiquilin 2 is not a UPR target gene in humans (Figure S2).

In a previous study it has been shown that ubiquilin 1 protein levels are reduced in early and late stages of AD [15]. Here we report that also ubiquilin 2 levels are decreased in the brain of AD patients. We do not find altered ubiquilin 2 mRNA levels in AD patients and thus, changes in ubiquilin 2 occur on a translational or posttranslational level. However, the decrease in ubiquilin 2 levels is not specific for neurons bearing hyperphosphorylated tau or tau inclusions, but rather appears to be associated with neurons free of pathological tau. Recently, ubiquilin 1 was found to accumulate in Hirano bodies in AD hippocampus and although no double immunohistochemistry was performed, stainings on serial sections suggest that also ubiquilin 1 does not colocalize with NFTs [24]. It was shown that ubiquilin 1 reduction results in an increase of amyloid precursor protein (APP) fragments [15] and more recently that ubiquilin 1 modulates γ-secretase cleavage [25]. It is possible that loss of ubiquilin 2 contributes to AD pathology via APP processing as well, but this requires further investigation.

In ALS, ubiquilin 2 mutations have been identified in families afflicted with ALS/dementia [2,15]. Because of the distinct ubiquilin 2 pathology identified in a variety of ALS cases with and without ubiquilin 2 mutations, the term ubiquilinopathy has been proposed [26]. ALS/dementia, tauopathies and other neurodegenerative dementias have in common inclusions that are immunoreactive for ubiquitin and the ubiquitin-binding protein p62 in the hippocampus and other brain regions [1]. In addition, impairment of the proteolytic systems is a common event in protein misfolding diseases. Ubiquilin 2 mediates protein degradation of ubiquitinated proteins and ubiquilin 2 dysfunction impairs protein degradation [2,15] and it was suggested that ubiquilin 2 pathology may be a general pathomechanism in neurodegenerative dementias. However, our results do not show evidence for a connection between ubiquilin 2 and tau pathology. Therefore this study indicates that different mechanisms underly the proteostatic disturbances in tauopathies and other proteinopathies.

## Supporting Information

Figure S1 **Ubiquilin 2 is diffusely distributed in the temporal cortex in tauopathies.** Immunohistochemical analysis of ubiquilin 2 in control (A) and AD (B) temporal cortex.
(PPT)

Figure S2 **UBQLN2 is not an ER stress-responsive gene in humans.** ER stress was induced in differentiated SK-N-SH cells using Tm (0.2 and 0.5 µg/ml) or 2-DG (20 mM) for 16h and mRNA levels were assessed by qPCR (shown are mean +SEM, n=3), eEF1A1 was used as a reference gene. Expression of the ER stress-responsive gene BIP is upregulated by all treatments (One-way ANOVA followed by Dunnett’s multiple comparison test, p <0.0001). Expression of ubiquilin 2 was only slightly increased with 2-DG and not changed upon treatment with Tm (One-way ANOVA followed by Dunnett’s multiple comparison test, p = 0.002). This indicates that human UBQLN2 is not an ER stress responsive gene.
(PPT)

Table S1 **Details of cases used for single staining in this study.**
(DOC)

Table S2 **Details of cases used for double staining in this study.**
(DOC)

Table S3 **Details of cases used for Western blotting in this study.**
(DOC)

Table S4 **Details of cases used for qPCR in this study.**
(DOC)
