# Supplementary figures and images for: Ubiquilin 2 Is Not Associated with Tau Pathology
Source: PLoS One. 2013 Sep 26;8(9):e76598. doi: 10.1371/journal.pone.0076598 (PMC3784422; doi:10.1371/journal.pone.0076598)

## Slide 1
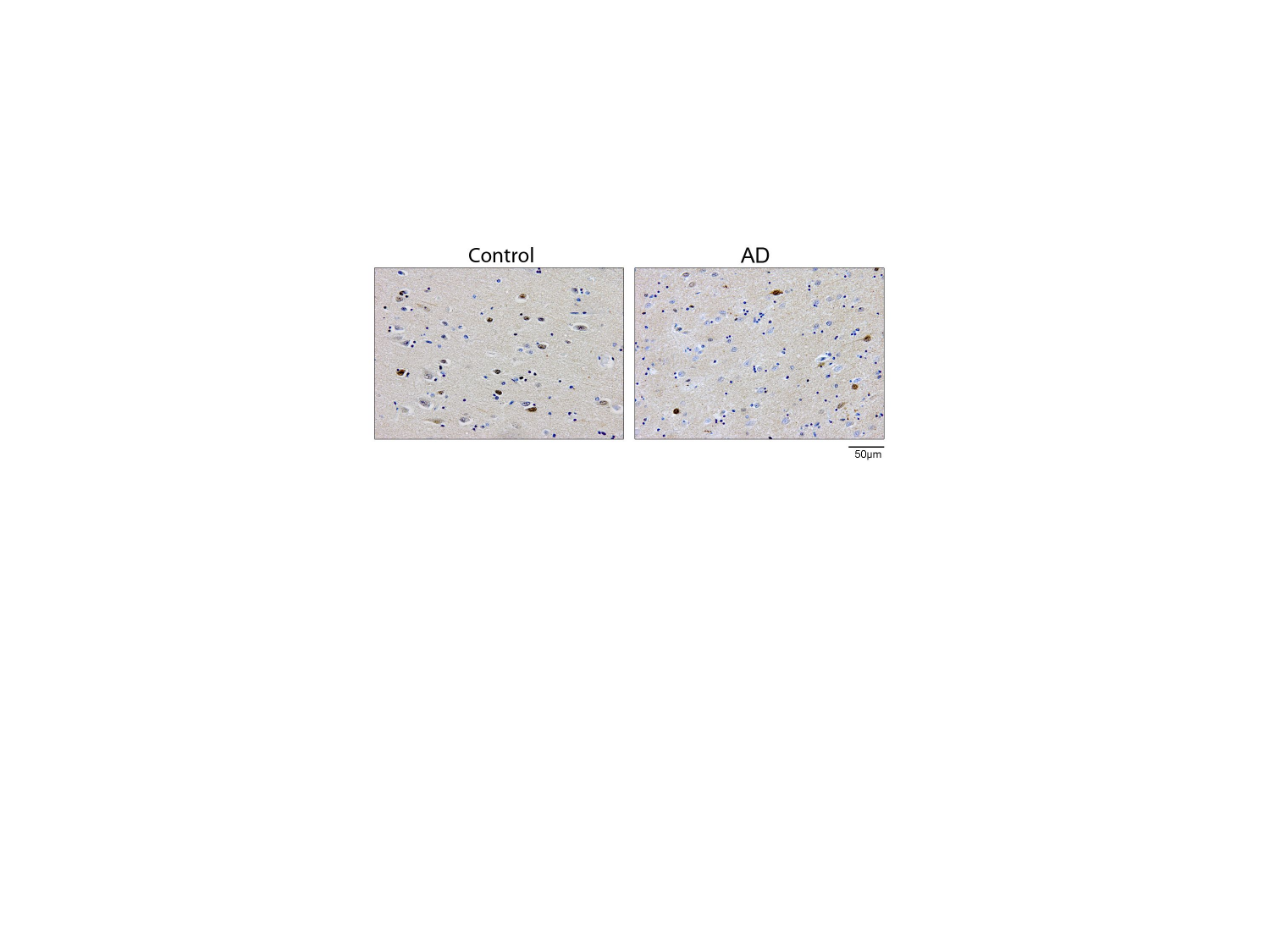

Supplement: Figure S1 — Ubiquilin 2 is diffusely distributed in the temporal cortex in tauopathies. Immunohistochemical analysis of ubiquilin 2 in control (A) and AD (B) temporal cortex. (PPT) [file pone.0076598.s001.ppt]

## Slide 1
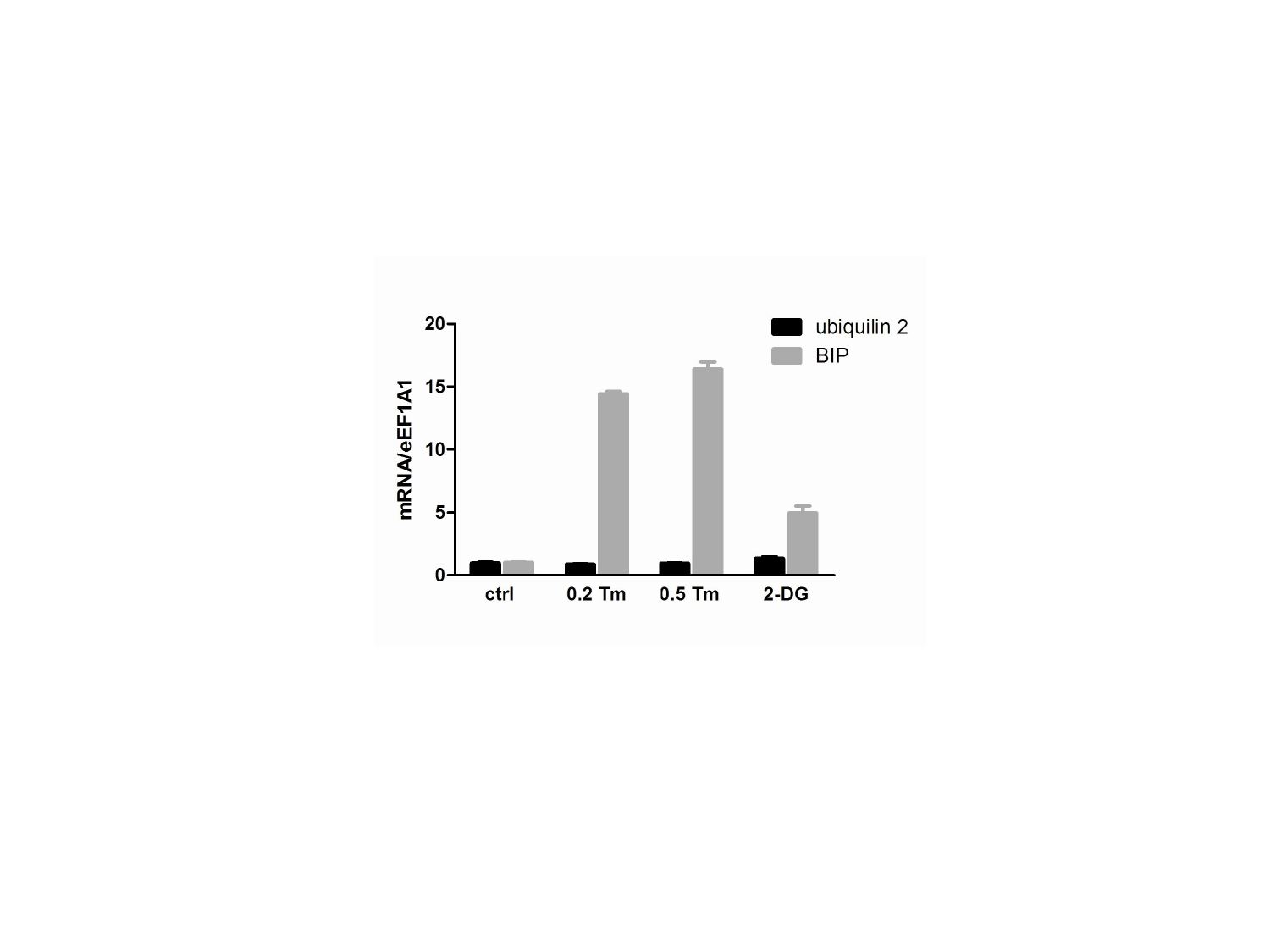

Supplement: Figure S2 — UBQLN2 is not an ER stress-responsive gene in humans. ER stress was induced in differentiated SK-N-SH cells using Tm (0.2 and 0.5 µg/ml) or 2-DG (20 mM) for 16h and mRNA levels were assessed by qPCR (shown are mean +SEM, n=3), eEF1A1 was used as a reference gene. Expression of the ER stress-responsive gene BIP is upregulated by all treatments (One-way ANOVA followed by Dunnett’s multiple comparison test, p <0.0001). Expression of ubiquilin 2 was only slightly increased with 2-DG and not changed upon treatment with Tm (One-way ANOVA followed by Dunnett’s multiple comparison test, p = 0.002). This indicates that human UBQLN2 is not an ER stress responsive gene. (PPT) [file pone.0076598.s002.ppt]
