# Supplementary material for: Ubiquilin 2 Is Not Associated with Tau Pathology
Source: PLoS One. 2013 Sep 26;8(9):e76598. doi: 10.1371/journal.pone.0076598 (PMC3784422; doi:10.1371/journal.pone.0076598)
Supplement: Table S1 — Details of cases used for single staining in this study. (DOC) [file pone.0076598.s003.doc]

**Table S1. Details of cases used for single staining in this study.**

| **Case** | **Pathological diagnosis** | **Gender** | **Age** | **Braak stage** |
| --- | --- | --- | --- | --- |
| 1 | con | f | 73 | 0 |
| 2 | con | m | 57 | 1 |
| 3 | con | m | 87 | 2 |
| 4 | con | m | 74 | 3 |
| 5 | AD | f | 87 | 5 |
| 6 | Familial AD | m | 57 | 6 |
| 7 | AD/LBV | f | 70 | 6 |
| 8 | AD | f | 78 | 5 |
| 9 | AD | m | 69 | 6 |
| 10 | AD/LBV | m | 75 | 4 |
| 11 | AD | f | 72 | 6 |
| 12 | PiD | f | 41 | - |
| 13 | FTD (MAPT G272V) | m | 51 | - |
| 14 | FTD (MAPT P301L) | f | 64 | - |
| 15 | PSP | f | 72 | 2 |
| 16 | FTD (MAPT P301L) | f | 66 | - |
| 17 | PiD | m | 57 | - |
| 18 | FTD (MAPT G272V) | m | 49 | - |
| 19 | FTD (MAPT L315) | f | 68 | - |
| 20 | PSP | f | 67 | 1 |
| 21 | PiD | m | 70 | - |

con, control; AD, Alzheimer’s disease; AD/LVB, Alzheimer’s disease with Lewy body variant pathology; FTD, frontotemporal dementia; MAPT, microtubule associated protein tau; PiD, Pick’s disease; PSP, progressive supranuclear palsy.
