# Supplementary material for: Ubiquilin 2 Is Not Associated with Tau Pathology
Source: PLoS One. 2013 Sep 26;8(9):e76598. doi: 10.1371/journal.pone.0076598 (PMC3784422; doi:10.1371/journal.pone.0076598)
Supplement: Table S3 — Details of cases used for Western blotting in this study. (DOC) [file pone.0076598.s005.doc]

**Table S3. Details of cases used for Western blotting in this study.**

| **Case** | **Pathological diagnosis** | **Gender** | **Age** | **Braak stage** | **PMI** |
| --- | --- | --- | --- | --- | --- |
| **WB1** | | | | | |
| 1 | con | f | 41 | 1 | 13:30 |
| 2 | con | m | 78 | 1 | 05:35 |
| 3 | con | f | 82 | 1 | 03:45 |
| 4 | con | f | 72 | 1 | 06:45 |
| 5 | AD | m | 80 | 6 | 04:20 |
| 6 | AD | f | 83 | 4 | 05:20 |
| 7 | AD | f | 70 | 6 | 04:30 |
| 8 | AD | f | 88 | 5 | 05:05 |
| **WB 2** | | | | | |
| 1 | con | f | 78 | 1 | 17:40 |
| 2 | con | f | 84 | 1 | 06:55 |
| 3 | con | f | 71 | 1 | 07:10 |
| 4 | AD | f | 85 | 6 | 05:10 |
| 5 | AD | m | 74 | 6 | 05:35 |
| 6 | AD | f | 72 | 6 | 05:55 |

PMI, post-mortem interval; WB, Western blot; con, control; AD, Alzheimer’s disease.
