# Supplementary material for: Ubiquilin 2 Is Not Associated with Tau Pathology
Source: PLoS One. 2013 Sep 26;8(9):e76598. doi: 10.1371/journal.pone.0076598 (PMC3784422; doi:10.1371/journal.pone.0076598)
Supplement: Table S4 — Details of cases used for qPCR in this study. (DOC) [file pone.0076598.s006.doc]

**Supplemental table 4. Details of cases used for qPCR in this study.**

| **Case** | **Pathological diagnosis** | **Gender** | **Age** | **Braak stage** | **PMI** |
| --- | --- | --- | --- | --- | --- |
| 1 | con | f | 81 | 1 | 06:40 |
| 2 | con | m | 85 | 1 | 04:15 |
| 3 | con | m | 80 | 0 | 07:15 |
| 4 | con | m | 84 | 1 | 07:05 |
| 5 | con | f | 91 | 3 | 08:00 |
| 6 | con | m | 87 | 2 | 07:20 |
| 7 | con | m | 74 | 3 | 05:00 |
| 8 | con | f | 86 | 2 | 06:25 |
| 9 | con | m | 84 | 1 | 07:05 |
| 10 | con | f | 93 | 2 | 05:50 |
| 11 | con | f | 85 | 1 | 05:00 |
| 12 | AD | f | 91 | 6 | 05:45 |
| 13 | AD | f | 81 | 6 | 06:00 |
| 14 | AD | f | 67 | 6 | 06:05 |
| 15 | AD | f | 75 | 5 | - |
| 16 | AD | m | 75 | 5 | 05:25 |
| 17 | AD | f | 94 | 4 | 05:00 |
| 18 | AD | m | 86 | 3 | 05:35 |
| 19 | AD | f | 94 | 5 | 04:30 |
| 20 | AD | f | 89 | 5 | 10:20 |
| 21 | AD | f | 86 | 4 | 05:55 |
| 22 | AD | f | 84 | 4 | 04:50 |

PMI, post-mortem interval; qPCR, quantitative PCR; con, control; AD, Alzheimer’s disease.
